# Outcomes of transvenous lead extraction in patients with lead perforation: A single‐center experience

**DOI:** 10.1002/clc.23327

**Published:** 2020-01-06

**Authors:** Xu Zhou, Feng Ze, Ding Li, Long Wang, Jihong Guo, Xuebin Li

**Affiliations:** ^1^ Department of Cardiac Electrophysiology Peking University People's Hospital Beijing China

**Keywords:** lead perforation, percutaneous subxiphoid pericardial puncture, pericardial effusion

## Abstract

**Background:**

Management of cardiac perforation caused by the lead of a cardiac implantable electronic device (CIED) is currently unclear. This study evaluated the outcomes of transvenous lead extraction (TLE) in patients with cardiac perforation caused by a transvenous lead.

**Hypothesis:**

Removal of perforated lead by transvenous approach is safe and effective.

**Methods:**

The medical records of all patients diagnosed with cardiac perforation by a pacing or defibrillator lead in Peking University People's Hospital from January 2008 to January 2019 were reviewed. We included patients who were managed by TLE.

**Results:**

A total of 53 patients (30 men; mean age: 67 ± 15 years) with lead perforation managed by TLE were included. Most of the perforated leads (94.9%) were pacemaker leads. Forty‐three leads (81.1%) were implanted within 1 year. Ten patients with a high risk of hemopericardium underwent percutaneous subxiphoid pericardial puncture prior to TLE. All 53 culprit leads were removed completely without major complications. Simple traction with or without a locking stylet was sufficient in 51 patients (96.2%). Forty‐eight patients (90.6%) had a new active‐fixation lead reimplanted. No patients showed evidence of new‐onset or worsening pericardial effusion during the procedure and hospital stay. During a median follow‐up time of 16 months, no recurrence of symptoms associated with lead perforation or CIED‐related infection were reported.

**Conclusion:**

In most patients with lead perforation, TLE can be a safe and effective management approach.

## 1 INTRODUCTION

Myocardial perforation is a serious but uncommon complication associated with the placement of a cardiac implantable electronic device (CIED). The reported incidences range from 0.1% to 0.8% for pacemaker leads and from 0.6% to 5.2% for implantable cardioverter‐defibrillator leads.^1^

The presenting symptoms and signs are variable and include chest pain, muscle or diaphragm stimulation, pericardial effusion, and lead malfunction; some patients are asymptomatic. The optimal management of lead perforation is still a matter of debate. Many cases managed by surgical repair have been reported, and surgical management is recommended by expert consensus.^2^ Recently, percutaneous lead extraction with surgical backup has been proposed as an alternative approach by some authors, although the data are still limited.^3^, ^4^, ^5^ The objective of this study was to determine the feasibility and safety of the percutaneous management of lead perforation.

## 2 METHODS

### 2.1 Study populations

The medical records of all patients diagnosed with cardiac perforation by a pacing or defibrillator lead in Peking University People's Hospital from January 2008 to January 2019 were reviewed. Patients who were managed by transvenous lead extraction (TLE) were included, and patients managed by a conservative approach (such as electronic programming) or an open‐heart lead extraction were excluded. Clinical characteristics, details of the device features, outcomes and complications related to TLE procedures, and follow‐up data were collected and analyzed.

Consistent with previous studies, cardiac perforation was suspected when patients presented with the following: (a) chest pain, dyspnea, and extracardiac muscle stimulation; (b) pericardial effusion; and (c) significantly altered electrical parameters.^6^, ^7^ The diagnosis of lead perforation was confirmed by conclusive imaging evidence (chest X‐ray, fluoroscopy, echocardiogram, or computed tomography [CT]) when necessary.

Acute lead perforation, which occurs during or shortly after implantation (<24 hours), may be hemodynamically unstable and always requires emergency treatment. Subacute (24 hours‐1 month) and delayed (>1 month) perforation are exceedingly rare and often asymptomatic. In the present study, acute and subacute perforations are collectively referred to as early perforation.

The study proposal was approved by the Ethics Committee of the Peking University People's Hospital. Informed consent was obtained from all patients.

### 2.2 Procedure descriptions

#### 2.2.1 Transvenous lead extraction

TLE was performed in the electrophysiology laboratory with continuous electrocardiographic and arterial blood pressure monitoring. A cardiac surgery team and an operating room were readily available. The procedure was performed under local or general anesthesia, depending on the patient's general health and physician's preference. Transesophageal echocardiography was performed in patients who were given general anesthesia. All perforating leads were extracted by a “stepwise” approach, which has been described previously.^8^ First, manual traction was attempted after retraction of the screw in the case of an active‐fixation lead. Second, if simple manual traction failed, an appropriately sized locking stylet was inserted into the lumen and locked at the distal part of the lead to maintain lead integrity, and the manual traction was repeated. Third, if manual traction with the locking stylet was still unsuccessful and the perforating lead was a pacing lead, a femoral approach with a Needle's Eye Snare and Femoral Workstation (Cook Medical, Bloomington, Indiana) was undertaken; if the targeted lead was a defibrillator lead, a laser‐powered sheath (SLS II sheath; Spectranetics, Colorado Springs, Colorado) was advanced over the lead to release fibrous adhesions from the site of vascular entry to the superior vena cava/right atrial junction.

Prior to attempted lead extraction, pericardiocentesis was performed and a pigtail catheter left in the pericardial sac in those patients with older culprit lead (>5 years) with passive fixation, preoperative pericardial effusion, or perforation beyond the pericardial sac.

#### 2.2.2 Reimplantation

During the same procedure, a new active‐fixation pacing or defibrillator lead was implanted at a different site if mandatory. For patients with CIED infection, the reimplantation was performed during another procedure. The pulse generator was replaced if the remaining charge was less than 5 years.

### 2.3 Procedure outcomes and complications

The definitions of outcomes and complications related to lead extraction are presented in the 2017 Heart Rhythm Society (HRS) consensus document.^9^

### 2.4 Post‐procedure evaluation and follow‐up

In all patients, serial daily clinical and echocardiographic assessment was performed to detect possible new‐onset or worsening pericardial effusion. For patients with preoperative drainage, the pigtail catheter was removed when the drainage was less than 40 mL per 24 hours. Chest X‐ray and device interrogation were performed before discharge.

Patients were scheduled to visit our department at 1, 3, 6, and 12 months. Telephone interviews were conducted if necessary. Recurrence of symptoms, pericardial effusion or lead perforation, development of CIED infection, and development of symptomatic pericarditis were defined as the endpoints. Death during the follow‐up period was also recorded.

### 2.5 Statistical analysis

Continuous variables were reported as mean ± SD and comparisons between groups were based on a two‐sample *t* test (parametric) or Wilcoxon rank‐sum test (nonparametric). Categorical variables were summarized as percentages and group comparisons were based on Fisher's exact test. A *P* value of less than .05 was considered significant. Statistics were analyzed with SPSS version 22 (SPSS, Chicago, Illinois).

## 3 RESULTS

### 3.1 Patients' characteristics and clinical features

Peking University People's Hospital is one of the largest referral centers for cardiac device extraction and pacing complications in China. A total of 67 patients with lead perforation were transferred to us from different regional hospitals from May 2008 to January 2018. Thirty‐five (52.2%) patients were from low‐volume centers with a procedure rate <50 per operator per year. Ten patients managed by open‐heart surgery and four patients managed by a conservative approach were excluded. Fifty‐three patients with lead perforation managed by TLE and lead reimplantation were included in our cohort. The mean (SD) age of the study patients, 56.6% of whom were female, was 67 (15) years (Table 1).

**Table 1:** Baseline characteristics of patients

Variables	All subjects (n = 53)
Age (y)	67 ± 15
Male sex	30 (56.6)
BMI (kg/m^2^)	23.9 ± 2.3
Clinical presentation	
Chest pain	31 (58.5)
Syncope	3 (5.6)
Dyspnea	6 (11.3)
Muscle stimulation	2 (3.8)
Asymptomatic	9 (20.8)
Comorbid conditions	
Hypertension	14 (26.4)
CAD	5 (9.4)
DM	2 (3.8)
NICM	1 (1.9)
AF	5 (9.4)
Renal dysfunction	4 (7.5)
Previous cardiac surgery	2 (3.8)
Medications	
Aspirin	12 (22.6)
Warfarin	5 (9.4)
Steroids	2 (3.8)
LVEF (%)	63 ± 10
Pericardial effusion	
Large	1 (1.9)
Moderate	3 (5.7)
Small	2 (3.8)
None	47 (88.6)
INR	1.2 ± 0.4
PLT (×10^9^/L)	253 (95)
Hb (g/dL)	12.1 (1.6)
Perforation categories	
Acute	9 (17.0)
Subacute	28 (52.8)
Delayed	16 (30.2)

*Note*: Data given as mean ± SD or n (%).

Abbreviations: AF, atrial fibrillation; BMI, body mass index; CAD, coronary artery diseases; DM, diabetes mellitus; Hb, hemoglobin; INR, international normalized ratio; LVEF, left ventricular ejection fraction; NICM, nonischemic cardiomyopathy; PLT, platelet.

Thirty‐seven patients (69.8%) were early perforations. Symptoms suggestive of lead perforation were reported in 42 patients (79.2%). Chest pain was the principal symptom, present in 31 patients (58.5%). Chest pain was more frequently reported in patients with early perforations compared with late perforations (70.3% vs 31.2%, *P* = .014). Nine patients (20.8%) were asymptomatic, and late perforations were more often found to be asymptomatic (50.0% vs 8.8%, *P* = .001). The median time from implant to symptom onset or diagnosis was 14 hours (range: 6‐19 hours) for the acute group, 7 days (range: 2‐30 days) for the subacute group, and 36 months (range: 1‐84 months) for the delayed group.

**Figure 1:**
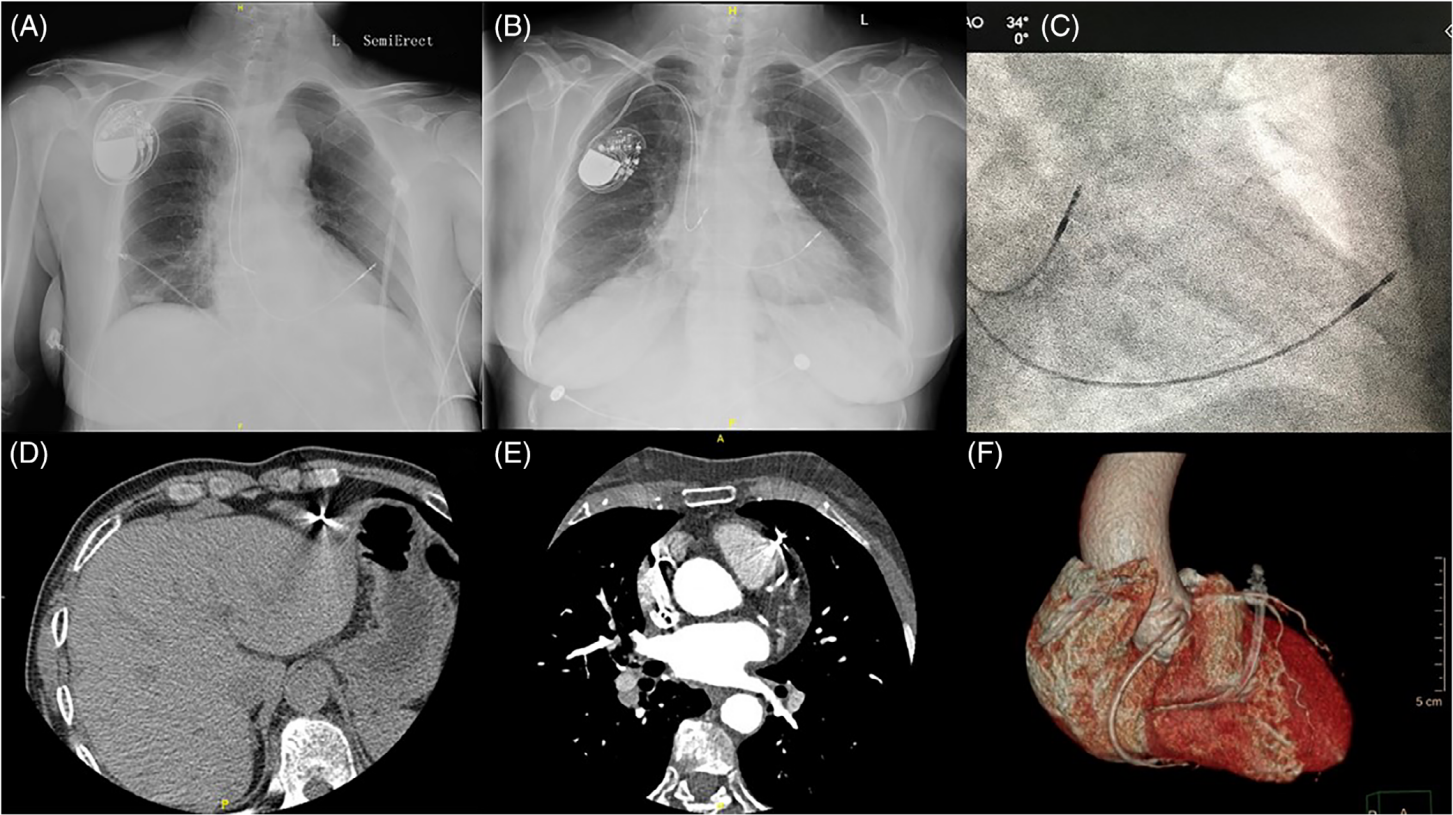
Imaging findings of lead perforation. A, Chest X‐ray demonstrating RV perforation by a PM lead. B, RV lead perforation suspected on AP chest X‐ray. C, RV lead perforation confirmed by fluoroscopy image (same patient as with B) showing lead dislocation outside the border of the heart. D, RV lead shown on chest CT. E and F, lead perforation confirmed by chest CT and corresponding reconstruction (AP view). AP, antero‐posterior; CT, computed tomography; PM, pacemaker; RV, right ventricular

Before lead extraction, all patients received a chest X‐ray, echocardiogram and device interrogation; if those imaging modalities were nondiagnostic, CT was performed. All diagnoses of lead perforation were confirmed by positive imaging tests (15 by CT, 20 by chest X‐ray, eight by fluoroscopy, and 10 by an echocardiogram; Figure 1).

### 3.2 Characteristics of CIEDs

The details of device types and perforated leads are compared in Table 2.

**Table 2:** Characteristics of perforated leads

Time from implantation	Number of perforated leads	Atrial leads	Lead malfunction	Extrapericardial lead tip	Pericardial effusion	Preoperative pericardiocentesis
<6 mo	30 (56.6)	5 (83.3)	7 (36.8)	1 (13.3)	1 (16.7)	1 (10)
6–12 mo	13 (24.6)	1 (16.7)	5 (26.3)	2 (66.7)	4 (66.6)	7 (70)
1–3 y	5 (9.4)	0 (0)	4 (21.1)	0 (0)	1 (16.7)	1 (10)
>3 y	5 (9.4)	0 (0)	3 (15.8)	0 (0)	0 (0)	1 (10)
Total	53	6	19	3	6	10

*Note*: Data are provided as n (%).

In our study, most of the perforated leads (51/53, 94.9%) were pacemaker leads. Of the 53 culprit leads, 41 (77.4%) were active‐fixation leads, twice as common as passive‐fixation lead perforation (12/53, 22.6%). The majority (43/53, 88.1%) of perforated leads had been implanted less than 1 year previously. Of the 47 (88.7%) right ventricle perforations, those at the apex were more frequent (35/47, 74.5%). Perforation beyond the pericardial sac was detected in three patients (3/53, 5.6%), all of whom had excessive loop of the lead left during implantation. Device interrogation showed abnormal lead parameters in 19 patients (35.8%), including three atrial leads and 16 ventricular leads. Six patients (11.4%) presented with pericardial effusion, all of whom had right ventricle perforation (Table 1).

### 3.3 Outcomes of TLE

Indications for TLE were chest pain in 31 patients (58.5%), continuous pericardial effusion in six (11.4%), extracardiac stimulation in two (3.8%), and lead malfunction that could not be repaired by electronic programming in 14 patients (26.3%). Compared with delayed perforation patients, the early perforation patients underwent a lead extraction procedure more frequently for mechanical complications (such as chest pain or muscle stimulation) and pericardial effusions (70.3% vs 43.8%, *P* = .02). Prior to attempted lead extraction, pericardiocentesis with pericardial drain insertion was carried out in 10 patients (18.9%): six with pericardial effusion, one with old (>5 years) passive‐fixation lead perforation (7 years), and three with lead perforation beyond the pericardial sac (to the pleural cavity in two and lung tissue in one). In patients with pericardial effusion before surgery, pericardial fluid was grossly bloody, with median drainage volume of 215 mL (interquartile range: 50‐450 mL). All procedures were performed in the electrophysiology laboratory. Sixteen patients (30.2%) were operated on under general anesthesia with transesophageal echocardiography monitoring. In local anesthesia cases, transthoracic echocardiography was performed.

All culprit leads were removed completely. Simple traction with or without a locking stylet was sufficient in 51 patients (96.2%), in whom the median dwell time was 14 days (range: 1‐1800 days). A Needle's Eye Snare was employed in both a 7‐year‐old and a 4‐year‐old passive‐fixation pacing lead. No permanent disabling complications or procedure‐related deaths occurred; consequently, the complete procedural success rate was 100%. All pericardial catheters had minimal drainage (<20 mL) during the procedure.

The clinical characteristics, device parameters, and procedural information of patients with preoperative pericardial drainage are summarized in Table 3.

**Table 3:** Clinical characteristics, device parameters, and procedure information of patients with prophylactic pericardial drainage

Patient#	Age	Gender	Device	Symptoms/signs	Time since implant	Fixation	Site of perforation	Anesthesia	Tools
1	88	Male	PM	Pericardial‐e	12 mo	Active	RV free wall	Local	Locking stylet
2	46	Male	PM	Chest pain	84 mo	Passive	RV free wall	General	Snare
3	64	Female	ICD	Pericardial‐e	6 mo	Active	RV apex	Local	Locking stylet
4	69	Male	CRT‐D	Pericardial‐e	8 mo	Active	RV apex	Local	Locking stylet
5	89	Male	PM	Pleural‐e	6 mo	Active	RV apex	Local	Locking stylet
6	64	Female	PM	Pericardial‐e	32 mo	Active	RV free wall	Local	Locking stylet
7	69	Male	PM	Lung perforation	8 mo	Active	RA	General	Locking stylet
8	74	Female	PM	Pleural‐e	12 mo	Active	RV apex	Local	Locking stylet
9	56	Female	PM	Pericardial‐e	8 mo	Active	RV apex	Local	Locking stylet
10	65	Male	PM	Pericardial‐e	10 d	Active	RV apex	Local	Locking stylet

Abbreviations: CRT‐D, cardiac resynchronization therapy‐defbrillator; ICD, implantable cardioverterdefbrillator; Pericardial‐e, pericardial effusion; Pleural‐e, pleural effusion; PM, pacemaker; RV, right ventricular; RA, right atrium.

### 3.4 Reimplantation

Forty‐eight patients (90.6%) had a new active‐fixation lead implanted in a different location than previously during the same or another procedure. The remaining five patients (9.4%) had no indications for reimplantation (four patients had no indications for pacing at the very beginning and pacemakers were implanted by mistake; one patient with an implant for tachy‐brady syndrome had developed persistent atrial fibrillation). One patient had pneumothorax during the reimplantation procedure and fully recovered after closed thoracic drainage.

### 3.5 Post‐procedure evaluation and follow‐up

After lead extraction and reimplantation, the symptoms resolved in all patients. After the procedure, no clinical signs suggestive of cardiac tamponade were detected, and echocardiography showed no new‐onset or worsening pericardial effusion during hospital stay. All pericardial catheters were removed within 24 hours after the procedure. All patients were discharged in a stable condition with satisfactory sensing and pacing parameters.

Follow‐up was available for 49 patients (92.5%). The median follow‐up duration was 16 months (range: 3‐20 months). During follow‐up, one patient died of multiple organ failure caused by severe pneumonia after 6 months, and another had the new ventricular lead extracted 13 months after the procedure because of an elevated pacing threshold. The remaining devices functioned well. No recurrence of symptoms associated with pericardial effusion or lead perforation was reported. There were no CIED‐related infections.

## 4 DISCUSSION

### 4.1 Clinical manifestation of lead perforation

Lead perforation is a rare complication after lead implantation, with most cases occurring early after implantation. Cardiac perforation can also occur several days or even months after implantation.^1^, ^10^, ^11^ In our cohort, more than 80% of the patients had subacute and delayed perforations. Cardiac perforations tend to be more prevalent in active‐fixation leads because the helical screwing mechanism is prone to penetration through the myocardium. In some case series, all culprit leads were reported to be active‐fixation leads.^4^, ^12^, ^13^ However, in the present study, of the 53 perforated leads, nearly 30% were passive‐fixation leads. Furthermore, a recent population‐based cohort study performed in Taiwan reported no difference in the risk of cardiac perforation between active‐ and passive‐fixation pacing leads.^14^ Given that the atrial myocardium is thinner than the ventricular myocardium, it is easy to speculate that atrium perforation is more frequent than ventricle perforation, which has indeed been confirmed by previous studies.^15^

Clinical presentations of lead perforation may vary widely, from totally asymptomatic to sudden cardiac death. Symptoms such as chest pain, dyspnea, and muscle stimulation are important clues to an accurate diagnosis. Abnormal sense or pacing parameters may also indicate lead perforation. However, symptoms may vary widely, and asymptomatic cardiac perforations are not uncommon.^15^ Moreover, normal parameters do not exclude a perforation. A small perforation may result in the cathode being proximal to the epicardium and the anode proximal to or within the endocardium, resulting in pacemaker function within the normal range. Because visualization of the lead tip is the key component of the diagnosis of cardiac perforation, imaging tests such as chest radiography and echocardiography have more accuracy. Chest CT is currently considered the most sensitive for diagnosis.^4^

### 4.2 Management strategy

The optimal treatment of lead perforation is still a matter of debate. The prevailing question in the management of myocardial perforation is whether to remove the lead and, if yes, to remove it percutaneously or surgically. According to the 2017 HRS expert consensus statement, lead extraction should be considered if lead perforation causes pain, pericardial bleeding, other significant symptoms, or device malfunction.^9^ However, in asymptomatic lead perforations with normal lead function, whether to extract it remains controversial. Previous studies indicate that extraction of a perforated lead that is otherwise functioning well and causes no symptoms does not appear to be necessary, especially for a chronically implanted lead.^15^ These patients are always diagnosed incidentally by imaging examination, and most cases do not result in electrophysiologic consequences. Expectant management should be the first choice. It is also suggested that, for an asymptomatic perforated lead with lead malfunction, electronic programming should also be considered prior to risky lead revision. Henrikson et al reported an asymptomatic atrial lead perforation with poor sensing and pacing parameters in a 66‐year old woman with a dual‐chamber pacemaker implanted for tachy‐brady syndrome, who was managed by reprogramming the device to VVIR mode.^16^ Of the 67 patients referred to our center, four asymptomatic patients (6.0%) with chronic lead perforation were managed successfully by electronic programming, all of whom had dual‐chamber pacemakers. Two patients implanted for sick sinus syndrome presented with ventricular lead perforations, and one implanted for intermittent complete atrioventricular block presented with atrial lead perforation. As the culprit leads were not crucial, the devices were reprogrammed to rate responsive pacing mode with atrial pacing and sensing,atrial pacing is inhibited when there is intrinsic P waves (AAIR) and rate responsive pacing mode with ventricular pacing and sensing, ventricular pacing is inhibited when there is intrinsic QRS waves (VVIR), respectively. These patients were doing well at the latest follow‐up. The remaining case concerned ventricular lead perforation in a 56‐year‐old man with a single‐chamber pacemaker implanted for complete heart block. The lead failed to capture the ventricle at the maximum output in the tip‐to‐can configuration. After tip‐to‐ring configuration was programmed, the culprit lead was restored to normal functionality and the symptoms were relieved. Perforation may result in the cathode being distant from the epicardium while the anode is proximal to or within the endocardium. In this case, the culprit lead might become functional again via lead ring pacing, which was also confirmed by our experience. During perforated lead extraction, after the culprit lead is freed we pace from the lead tip or ring to estimate which electrode is still in contact with the heart muscle. Of the 19 nonfunctional leads, two atrial leads and 10 ventricular leads were able to capture the myocardium again. It could be speculated that if the ring electrode was available as the cathode over the long term, affected patients might have cardiac capture restored and surgery might be unnecessary. Similarly, Biffi et al reported that programmability of the sensing channel and configuration in CRT‐D should become available to repair right ventricular leads with sensing issues without reintervention.^17^ Therefore, more intensive programmability should be engineered in CIEDs, which may infrequently mandate lead revision or pocket opening to ensure permanent normal function of devices.

However, delayed perforation is not necessarily asymptomatic or without lead malfunction. In our cohort, 10 patients (11.9%) had a perforated lead older than 1 year. All of these patients had symptoms or lead malfunction that could not be repaired by electronic programming, which might be the reason why they were transferred to us from regional hospitals. For such patients, removal of the perforated lead may be the only solution.

According to a consensus endorsed by the American Heart Association, surgical removal of the perforated leads should be the preferred strategy.^2^ Many authors have reported cases of lead perforation managed successfully by a surgical approach.^18^, ^19^, ^20^ Alternatively, many case series (with numbers ranging from 3 to 31) suggest that the perforated lead may be safely removed percutaneously with surgical backup, consistent with our current results.^1^, ^3^, ^4^, ^12^, ^21^, ^22^ The complete procedural success rate reported in these series was 92% to 96%, which is comparable with our reported rate. Most of the perforated leads were removed by simple extraction because the dwell time was usually not overlong. In the case of a chronically implanted target lead, advanced extraction tools may be employed.^23^

In cases of lead perforation that has migrated beyond the pericardial space or into another cavity, such as the pleura, the peritoneal cavity and visceral organs, the digestive tract, and the intercostal muscles, surgical extraction seems to be the safest option to repair the site of perforation and injury to the adjacent structures at the same time.^24^, ^25^, ^26^ However, Archontakis et al recently reported four lead perforations beyond the pericardial space (diaphragm or abdominal cavity) that were treated percutaneously without major complications.^27^ In our study, three patients with a lead migrating into the pleural cavity or lung tissue were managed uneventfully with a percutaneous approach. However, given the small patient cohort in these studies, no definitive conclusions could be drawn and more studies are needed.

One of the most life‐threatening complications during percutaneous extraction of the perforated lead is cardiac tamponade. In the present study, no cardiac tamponade was detected during or after the procedure, in line with previous studies.^3^, ^4^ Myocardial “self‐sealing” properties, low pressure in the right ventricle, and fibrous tissue formed at the perforating site are beneficial to hemostasis of the perforated myocardium. As we know, the atrial myocardium is thinner and consists of fewer myocytes. For this reason, atrial perforations may have less ability to spontaneously close the perforated site and are more prone to pericardial effusion during or after the procedure. In the present study, neither atrial nor ventricular perforation led to new‐onset or worsening cardiac effusion, although the number of atrial perforations was small (only six patients). However, some reports have described cardiac tamponade during or shortly after removal of the perforated leads. Laborderie et al reported one case of cardiac tamponade among 10 patients with late right ventricular perforation treated with percutaneous lead extraction by simple traction.^21^ Huang et al reported three cases of cardiac tamponade that developed 1 to 4 days after the percutaneous lead removal procedure.^12^ After prompt recognition of cardiac tamponade, all patients recovered well after urgent pericardiocentesis, with no surgical repair being necessary. Therefore, transesophageal monitoring during the procedure and echocardiographic monitoring after the procedure are strongly recommended. Anticoagulation and antiplatelet therapy may be a risk factor for delayed cardiac tamponade.^12^, ^28^ Close monitoring of the international normalized ratio level is recommended. Anticoagulant agents should be discontinued before the procedure to achieve hemostasis.

Of note, we accessed the pericardial sac prior to the extraction procedure in patients with the following: perioperative pericardial effusion, perforation of old lead (>5 years) with tined electrodes (with more adhesion), and perforation beyond the pericardiac sac (a through‐and‐through perforation rather than partial perforation). Although these characteristics were assumed to be risk factors for pericardial effusion or cardiac tamponade after the culprit lead was removed, no pericardial effusion was detected. Therefore, preoperative pericardial drainage without cardiac effusion or tamponade is not mandatory and should not be used as a routine approach.

Owing to the “high‐risk” nature of the operation, the whole procedure should be performed in a center specializing in percutaneous lead extraction, under careful hemodynamic and echocardiographic monitoring. Following extraction, the patient should also be closely monitored, both clinically and by echocardiography, because of the risk of pericardial perfusion relapse.

In this series of 53 patients, there were no complications and no need for any action by surgery. However, we consider that surgical backup is necessary for these cases because they might result in uncontrolled bleeding, which requires a surgical approach. When pericardial drainage is ineffective in restoring blood pressure and there is evidence of significant continued bleeding, which could happen because of differences in the size and nature of the perforation, surgery must be rapidly performed.^28^ While surgical backup was not needed in our series, it is a critical component of these cases and is required to ensure an optimal outcome. Since urgent pericardial drainage can “buy” time until surgery, there is no need for a cardiothoracic surgeon to scrub in, although rescue thoracotomy should be performed promptly before it is too late.

### 4.3 Limitations

The present investigation has several limitations. First, this was a retrospective study at a single center. Second, the patients who were referred for surgical lead revision or managed conservatively were excluded because the aim of the study was to evaluate the outcomes of the percutaneous management approach. Third, the lead types were not evenly distributed because most of the perforated leads were ventricular leads and only two defibrillator leads were included.

## 5 CONCLUSION

Our study showed that lead perforation may occur relatively late after CIED implantation. Both active‐ and passive‐fixation leads can cause perforation. Chest pain, dyspnea, or altered electrical parameters after CIED implantation must prompt a radiologic and echocardiographic evaluation. For asymptomatic patients with lead malfunction, fully electronic programming should be considered prior to deciding on surgery. When lead revision is mandatory, most patients can be managed safely and effectively by TLE. While not needed in our series, surgical backup is a critical component to ensure an optimal outcome.

## CONFLICT OF INTEREST

The authors declare no potential conflict of interests.
